# Temperature Sensitivity and Basal Rate of Soil Respiration and Their Determinants in Temperate Forests of North China

**DOI:** 10.1371/journal.pone.0081793

**Published:** 2013-12-10

**Authors:** Zhiyong Zhou, Chao Guo, He Meng

**Affiliations:** 1 Ministry of Education Key Laboratory for Silviculture and Conservation, Beijing Forestry University, Beijing, China; 2 The Institute of Forestry and Climate Change Research, Beijing Forestry University, Beijing, China; 3 College of Forestry, Inner Mongolia Agriculture University, Hohhot, China; Tennessee State University, United States of America

## Abstract

The basal respiration rate at 10°C (R_10_) and the temperature sensitivity of soil respiration (Q_10_) are two premier parameters in predicting the instantaneous rate of soil respiration at a given temperature. However, the mechanisms underlying the spatial variations in R_10_ and Q_10_ are not quite clear. R_10_ and Q_10_ were calculated using an exponential function with measured soil respiration and soil temperature for 11 mixed conifer-broadleaved forest stands and nine broadleaved forest stands at a catchment scale. The mean values of R_10_ were 1.83 µmol CO_2_ m^−2^ s^−1^ and 2.01 µmol CO_2_ m^−2^ s^−1^, the mean values of Q_10_ were 3.40 and 3.79, respectively, for mixed and broadleaved forest types. Forest type did not influence the two model parameters, but determinants of R_10_ and Q_10_ varied between the two forest types. In mixed forest stands, R_10_ decreased greatly with the ratio of coniferous to broadleaved tree species; whereas it sharply increased with the soil temperature range and the variations in soil organic carbon (SOC), and soil total nitrogen (TN). Q_10_ was positively correlated with the spatial variances of herb-layer carbon stock and soil bulk density, and negatively with soil C/N ratio. In broadleaved forest stands, R_10_ was markedly affected by basal area and the variations in shrub carbon stock and soil phosphorus (P) content; the value of Q_10_ largely depended on soil pH and the variations of SOC and TN. 51% of variations in both R_10_ and Q_10_ can be accounted for jointly by five biophysical variables, of which the variation in soil bulk density played an overwhelming role in determining the amplitude of variations in soil basal respiration rates in temperate forests. Overall, it was concluded that soil respiration of temperate forests was largely dependent on soil physical properties when temperature kept quite low.

## Introduction

CO_2_ emission from soil and plants to the atmosphere determines the amplitude of feedbacks of forest ecosystems to global climate change. Accurate prediction of the amount of CO_2_ respired by forest soil is of great importance in evaluating the carbon balance of forest ecosystems. In most cases, soil respiration rate at a given temperature can be estimated by the empirical functions using soil basal respiration rate (R_10_, soil respiration rate at 10°C) and the temperature sensitivity of soil respiration (Q_10_, a proportional change in soil respiration with a 10°C increase in temperature) [1], [2], [3]. Therefore, it seems vital to identify the biophysical variables driving these two parameters to advance the research on soil carbon turnover.

Soil respiration is mostly controlled by soil temperature [3], [4], secondarily by soil moisture, nutrients [5], vegetation type [6], tree species composition [7], topography, and climate [8]. To increase the comparability of soil respiration rate under different environmental conditions, a standardized parameter (e.g. R_10_) is proposed when emphasizing the effects of biophysical factors other than temperature. Although soil basal respiration may also be influenced by the similar variables mentioned above [9], it is still of importance to make clear the relationship of soil basal respiration with biophysical variables in improving the precision of simulation models. This is because, for a specific forest ecosystem, some biophysical factors can be considered as additional predictive variables when estimating soil respiration rate using empirical methods [3], [10].

Great effort has been exerted to the response of soil respiration to a change in temperature in recent decades [2], [11], which is denoted in most studies to be the temperature sensitivity of soil respiration, and is theoretically represented by an invariant coefficient (Q_10_) of ∼2, especially in coupled climate-carbon cycle models [12], [13]. The extensive use of a fixed Q_10_ has brought large convenience in calculating the amount of CO_2_ respired from soil, but it has also evoked a controversy between theoretical studies and incubation experiments or field measurements [14]. It is demonstrated that the temperature sensitivity of soil respiration (Q_10_) can be influenced in ecosystems by many biophysical or physicochemical factors, including the forest floor conditions [15], soil physical properties [16], soil nutrients [17], and vegetation type [18]. Therefore, the Q_10_ originated from the temperature dependence equation shows distinct intersite difference or temporal variation [16], [17], [18]. Obviously, the application of a constant Q_10_ can not lead to an unbiased estimation of soil respiration rate for the studying ecosystem type any more.

Being illustrated by the calculation process of the common empirical function, an inherent correlation apparently exists between basal soil respiration and the temperature sensitivity [1], [19]. Mathematically, Q_10_ is dependent on, and acts as a multiplier of R_10_ [19]. Any effort paid on the single parameter has limited use in improving the estimating precision of the extensively applied empirical functions.

Temperate forests in northern China mainly extend along the mountain ridge with heterogeneous growing conditions, which provide a natural experimental place for continuing similar research work on model parameters of soil respiration. In this study, we investigated the instantaneous rate of soil respiration and environmental variables at a representative catchment of the temperate forests in China, and calculated the two model parameters using the temperature dependent function. Herein hypotheses were proposed that the apparent temperature sensitivity of soil respiration could display detectable variations among forest types with different micro-environmental properties, and biophysical variables other than soil temperature could play an important role in determining soil basal respiration rate when the temperature decreased to a comparatively low level. Accordingly, our main objectives of this paper were to: 1) quantify the changing magnitude of model parameters of soil respiration within or between forest types; 2) identify the predominant variables controlling the spatial heterogeneity of the two parameters on the catchment scale in temperate forests.

## Materials and Methods

### Ethics statement

This research was conducted on field sites with the permission of the Taiyueshan Long-Term Forest Ecosystem Research Station. We declare that no privately owned land was used in this study, and that the field investigation did not involve any protected or endangered plant and animal species, and that no human or animal subjects were used in this study. The research has adhered to the legal requirements of China during the field study period.

### Study site and experimental layout

This study was carried out at the catchment named after Xiaoshegou near the Taiyueshan Long-Term Forest Ecosystem Research Station (latitude 36°04′N, longitude 112°06′E; elevation 600 – 2600 m a.s.l), which is about 190 km southwest of Taiyuan in Shanxi province of China. Annual mean temperature varies between 10°C and 11°C, with 26°C in the warmest month of July and −23°C in coldest month of January; whilst mean annual precipitation ranges from 500 mm to 600 mm [20]. The hill in the study area is at an elevation of 1800 m with its bottom of 1200 m a.s.l. The soil type of the hill slope belongs to a Eutric Cambisols (FAO classification) or a Cinnamon soil (Chinese classification) with the mean soil depth of 30 cm to 50 cm, soil organic carbon content (SOC) of 0.77% to 5.47%, total nitrogen content (TN) of 0.036% to 0.232%, and soil pH from 6.9 to 7.6. The proportion of <0.01 mm and <0.001 mm soil fraction varies within the range of 46.54% to 63.10% and of 18.88% to 41.45%, respectively [21]. The dominant tree species in the forests are *Pinus tabuliformis*, *Quercus wutaishanica*, *Betula dahurica*, *Larix gmelinii var. principis-rupprechtii*, *Tilia mongolica*. The understory shrub community mainly consists of *Corylus mandshurica*, *Corylus heterophylla*, *Acer ginnala*, *Lespedeza bicolor*, *Philadelphus incanus*, *Rosa bella*, *Lonicera chrysantha*. The herbaceous community is commonly composed of *Carex lanceolata*, *Spodiopogon sibiricus*, *Rubia chinensis*, *Thalictrum petaloideum*, *Melica pappiana*.

Twenty 20×20 m plots spread along four hill ridges with different topography at the small catchment, including 9 broadleaved forest stands and 11 mixed conifer-broadleaved forest stands. The forest type was classified by the basal area ratio of coniferous to broadleaved tree species. The forest community was classified as the mixed forest type when its ratio fell within the range of 20% to 80%. Forest community structure was investigated in later Aug-2009. Each plant with diameter at breast height (DBH) >5 cm was measured for values of DBH and height respectively basing on tree species for these 20 plots. On each plot, five 5×5 m subplots were established for the investigation of shrub community, and five 1×1 m subplots for herbaceous community.

### Measurements of soil respiration

Soil respiration rate was measured once per month for each forest stand during the growing season of May to November in 2008 and 2009, using a Li-Cor infrared gas analyzer (LI-8100, Li-Cor Inc., Lincoln, NE, U.S.A.) equipped with a portable chamber. The chamber was put on the top of installed collars for 2 minutes before measurements. In early April, nine polyvinyl chloride (PVC) collars were evenly placed on each plot with eight collars arranged in a circle at 5 m to the plot center and one right at the center. The PVC collar of 10 cm in diameter and 5 cm in height was permanently inserted 3 cm into the soil with 2 cm remaining above the surface of the forest floor. The live herbs or seedlings were carefully removed out the collars to avoid bias due to its respiratory activity just after plant growth occurred. Concurrently, soil temperature at 10 cm depth adjacent to each PVC collar was monitored using a thermocouple probe attached to LI-8100 system. The averaged data of soil respiration and soil temperature across the nine PVC collars per month were fitted to the following exponential model [1], [10] to calculate basal parameters of soil respiration for each forest plot.
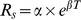
(1)where *R_s_* is in situ soil respiration rate measured in the field, *α* and *β* are model parameters, *T* is the measured soil temperature. According to equation (1), the temperature sensitivity of soil respiration was calculated by:
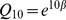
(2)


Soil basal respiration was calculated by:
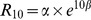
(3)


### Measurements of environmental variables

Shrub community was investigated by species for plant density and biomass of a representative sampling plant. The sampling plants were harvested and brought back to laboratory, and oven dried at 75°C to constant weight. The biomass of each shrub species within the community was estimated basing on plant density and its mean weight. The herbaceous plants in the 1×1 m subplot were all harvested for aboveground components. Additionally, litter on the forest floor was also collected in five 30×30 cm subplots on each plot. The herbaceous plant samples and litter were separately placed in envelope, transported to laboratory, and oven dried at 75°C for at least 48 h before weighing.

Soil cores of 4 cm in diameter and 20 cm in depth were sampled at five measurement points on each plot in later growing season of 2009. The air dried soil samples were mound to pass a 0.2 mm sieve for nutrient analysis after visible litter segments were picked out by hand. SOC and TN were determined separately following the modified Mebius method [22] and the Kjeldahl digestion procedure [23]. Soil phosphorus (P) was measured using the colorimetric determination method described by John [24]. Soil pH was measured in deionized H_2_O using Sartorius AG (PB-10, Sartorius, Germany), after equilibration for 1 h in a water: soil ratio of 2.5∶1. Soil cores were additionally excavated by a cylindrical sampler of 100 cm^3^ at five sampling positions on each plot, and oven dried at 110°C for at least 48 h in laboratory to measure the soil bulk density.

### Data analyses

Soil physicochemical properties were also averaged for each plot when their effects on R_10_ and Q_10_ were analyzed. A two-tailed t-test was applied to detect the differences of R_10_ and Q_10_ between these two forest types at α = 0.05. The spatial variability was expressed using the coefficient of variation (CV) calculated as the following.
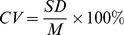
(4)where *SD* means standard deviation, and *M* represents mean value.

All these data analyses were carried out using the software of SPSS 15.0. Figures were made using the software of SigmaPlot in version 10.0.

In order to test the combined contribution of biophysical variables to the variability of R_10_ and Q_10_, redundancy analysis (RDA) [25] was conducted with R_10_ and Q_10_ as dependent variables and with selected biophysical variables, i.e. DBH, soil pH, variances in soil bulk density, soil TN and soil pH, as explanatory variables. RDA was performed using the software of Canoco for Windows 4.5.

## Results

### Inter- and intra-forest-type variations in basal parameters of soil respiration

R_10_ and Q_10_ were on average 10% and 11% higher in the broadleaved forest stands than in the mixed forest stands, although no statistically significant difference was detected between these two forest types (*P* = 0.25 for R_10_ and 0.91 for Q_10_). There existed large spatial heterogeneity in temperature sensitivity and basal rate of soil respiration among forest stands. The CV of R_10_ ranged from 11% in the broadleaved forest to 19% in the mixed forest, and the CV of Q_10_ varied from 24% in the broadleaved forest to 29% in the mixed forests (Table 1).

**Table 1:** Variation in basal parameters of soil respiration within or between forest types.

	R_10_ (µmol CO_2_ m^−2^ s^−1^)	CV of R_10_ (%)	Q_10_	CV of Q_10_ (%)
Broadleaved forest	2 a	11	4 a	24
Mixed forest	1 a	19	3 a	29

The significance of differences of basal parameters between forest types were separately tested by independent t - test (two - tailed) at α = 0.05 (n = 9 in the broadleaved forest, and 11 in the mixed forest). Same lowercase letter means no significant difference is detected at α = 0.05 within 95% confidence interval between the two forest types.

**Figure 1.Trends:**
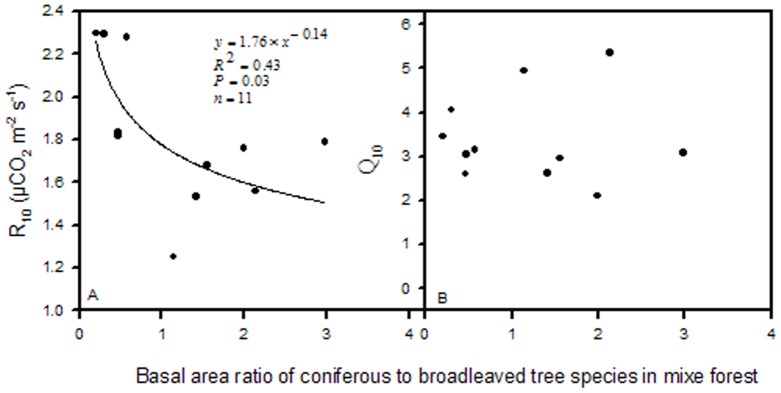
Trends of R_10_ and Q_10_ with basal area ratio of coniferous to broadleaved tree species.

Particularly, in the mixed forest stands, R_10_ was significantly affected by the basal area ratio between coniferous and broadleaved tree species, and greatly declined with the percentage of coniferous tree species. No significant correlation was found between Q_10_ and the basal area ratio in mixed forest stands (Fig. 1).

### Determinant variables of soil basal respiration rate

**Figure 2:**
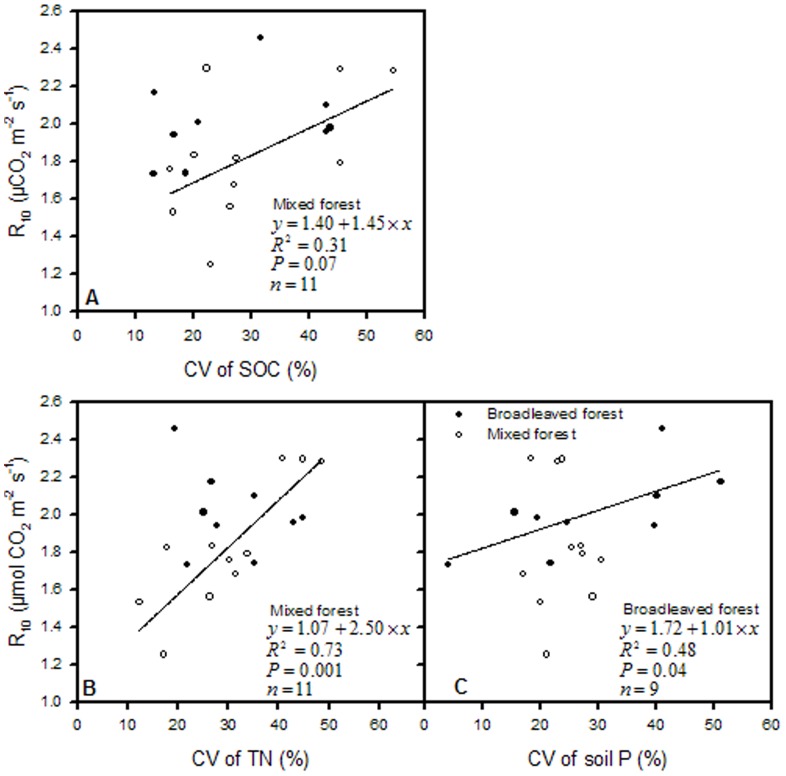
Correlations of R_10_ with the variations of SOC, soil TN, and soil P in broadleaved and mixed forests.

**Figure 3:**
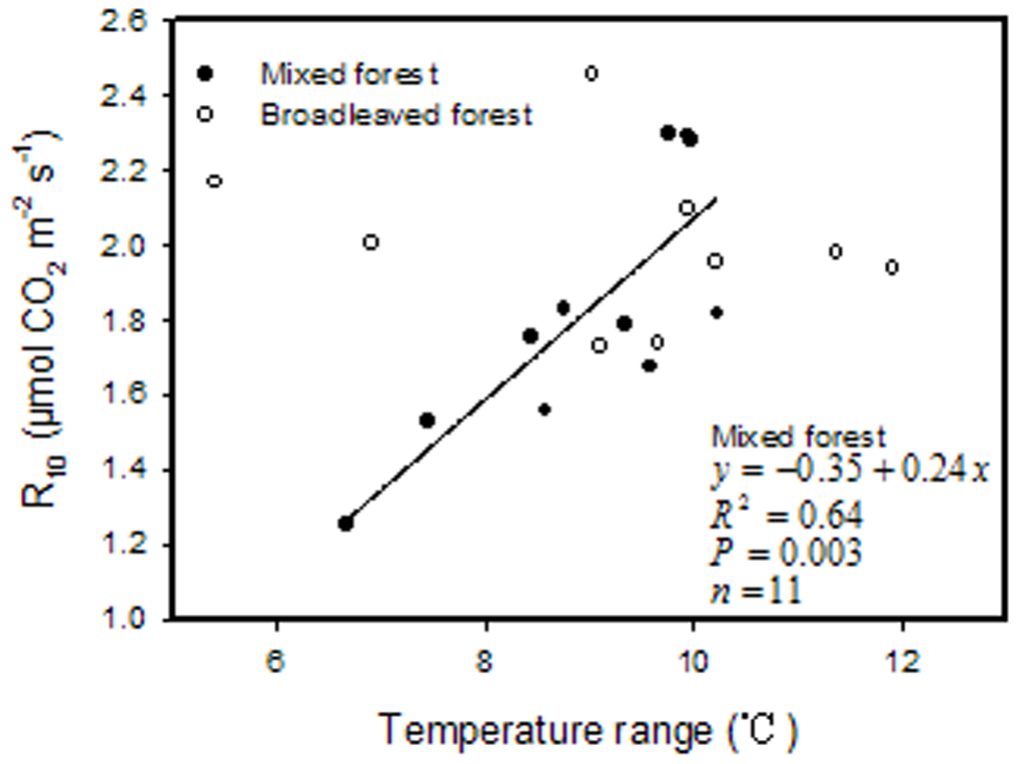
Correlations of R_10_ with soil temperature range for both forest types.

**Figure 4:**
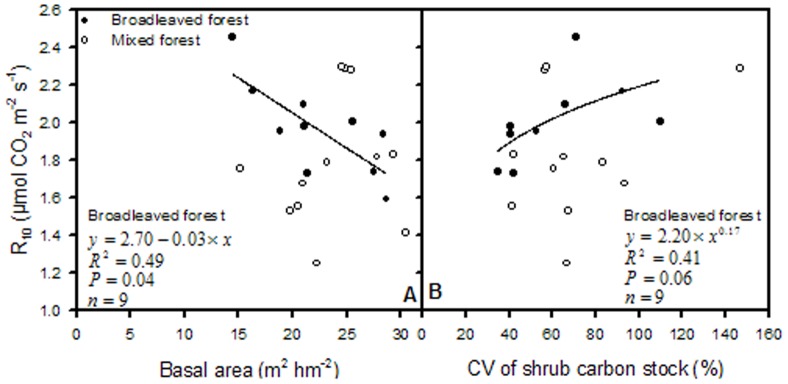
Correlations of R_10_ with basal area and CV of shrub carbon stock separately for both forest types.

R_10_ was mainly influenced by soil nutrient content and rose linearly with CV of SOC and CV of TN; soil temperature range during which soil respiration was monitored was significantly correlated with R_10_ in the mixed forest (Fig. 2A, 2B, and 3). Contrarily, in the broadleaved forest stands, R_10_ was largely determined by the basal area and the spatial variations of shrub carbon stock and soil phosphorus content (Fig. 2C and 4). There was a linearly inverse relationship between R_10_ and the basal area, whereas R_10_ increased differentially with increasing variations in shrub carbon stock and soil P in the broadleaved forest stands.

### Determinant variables of the temperature sensitivity of soil respiration

**Figure 5:**
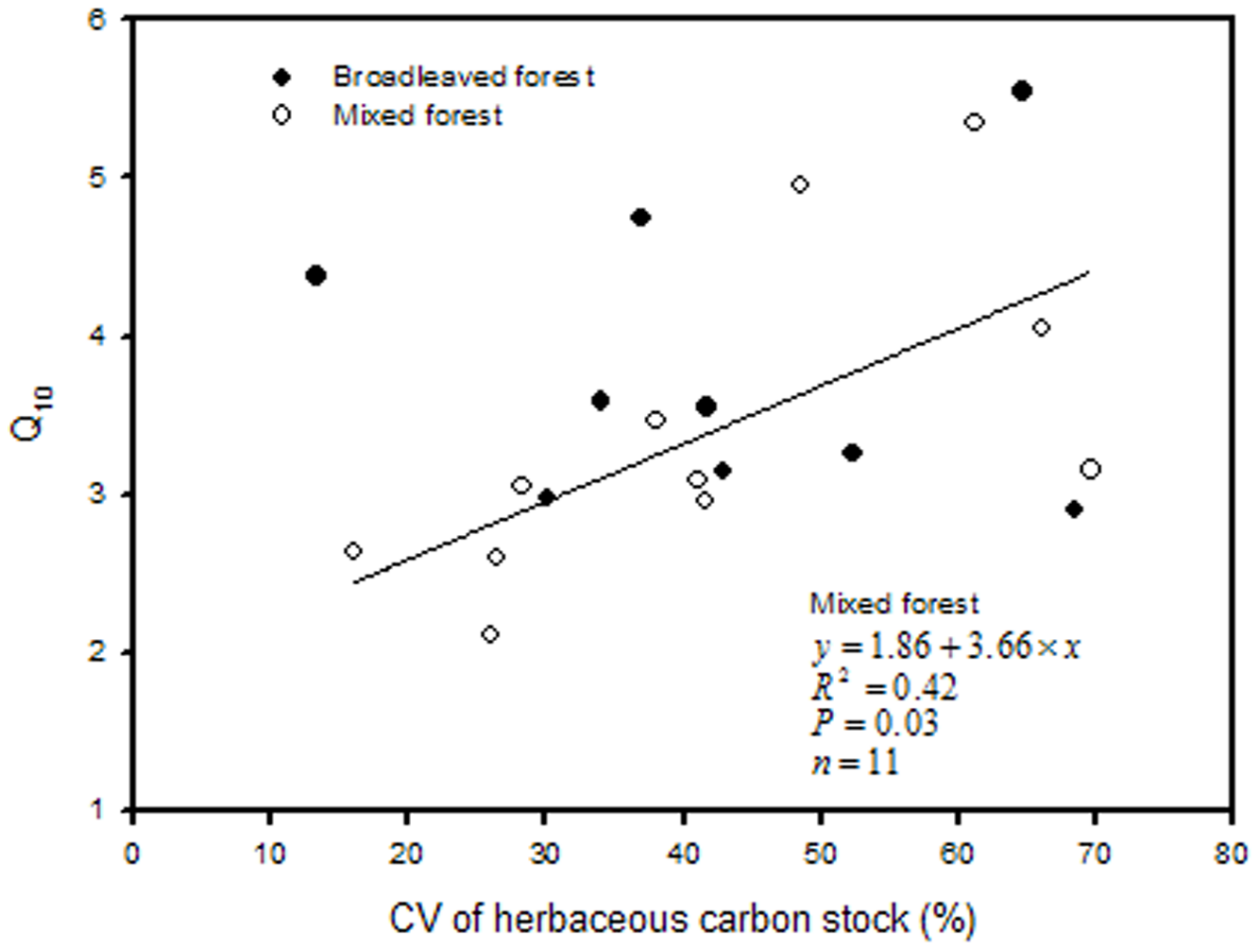
Relationships of Q_10_ with variation of herbaceous carbon stock in both forest types.

**Figure 6:**
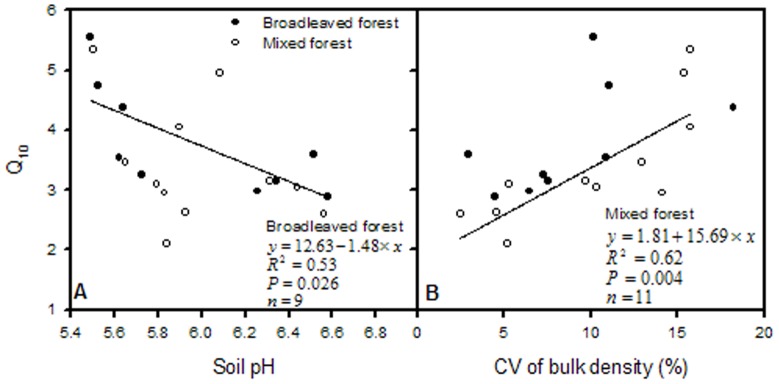
Relationships of Q_10_ with soil physical factors separately in broadleaved and mixed forest types.

**Figure 7:**
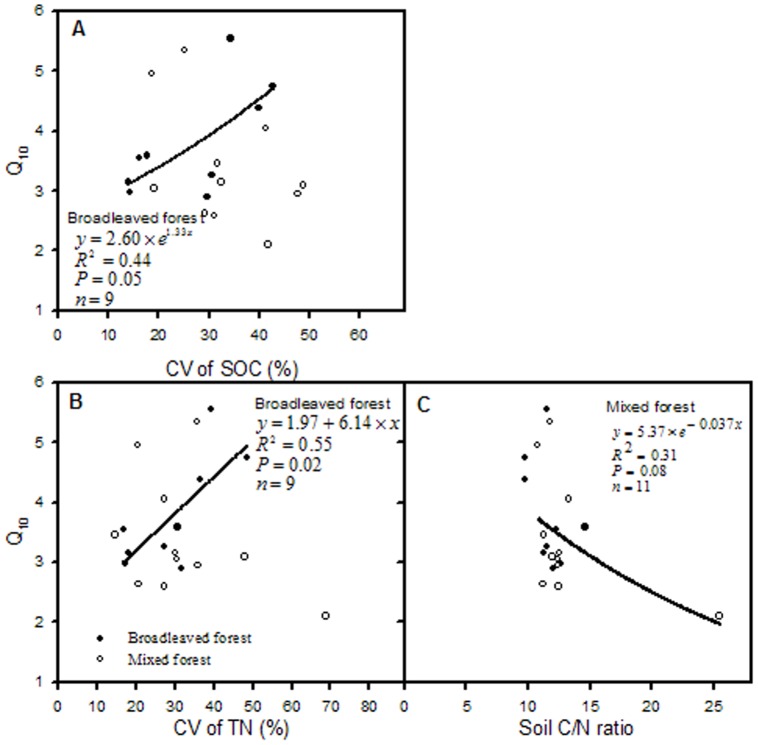
Relationships between soil chemical properties and Q_10_ respectively in broadleaved and mixed forest types.

In the mixed forest, Q_10_ was positively correlated with CV of herbaceous carbon stock and CV of soil bulk density (Fig. 5 and 6B), and negatively with soil C/N ratio (Fig. 7C). In the broadleaved forest, Q_10_ notably decreased with soil pH (Fig. 6A), but significant positive correlations were found between Q_10_ and CV of SOC and TN (Fig. 7A and B).

### Combined relationships among R_10_, Q_10_ and biophysical factors

**Figure 8:**
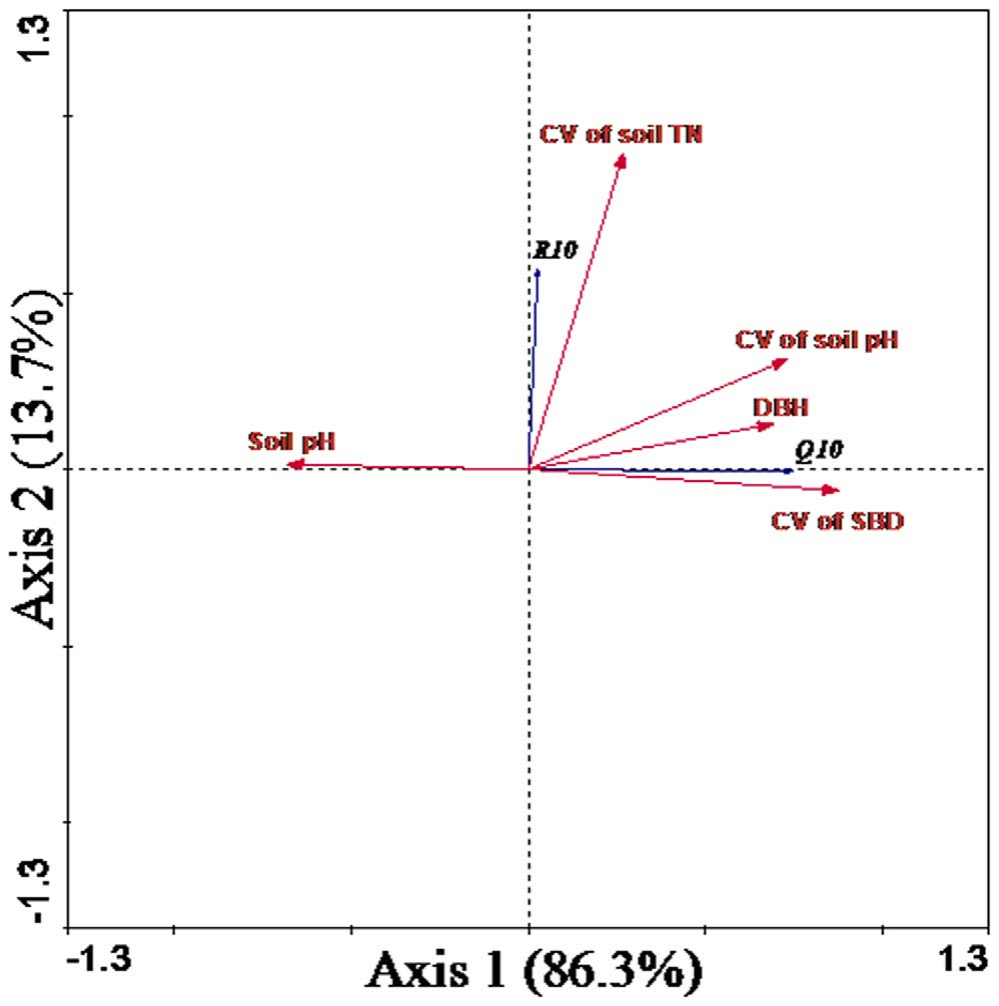
Redundancy analysis (RDA) among Q_10_, R_10_ and the biophysical variables. DBH means diameter at breast height; SBD means soil bulk density.

Although many environmental factors were found in this study to independently exert significant effects on individual parameter of R_10_ or Q_10_, 51% of the variations in both R_10_ and Q_10_ on the spatial scale were explained jointly by five biophysical variables, i.e., CV of soil bulk density, DBH, CV of soil TN, soil pH, and CV of soil pH, after forward selection of environmental variables. Particularly, most of the variations in R_10_ and Q_10_ were mainly ascribed to the variance of soil bulk density (Table 2). In addition, the importance of these selected factors was also highlighted by the result of Redundancy analysis, which showed that Axis 1 and Axis 2 accounted for 86.3% and 13.7% of the total variance in basal parameters of soil respiration, respectively (Fig. 8).

**Table 2:** The effects of biophysical variables on R_10_ and Q_10_ analyzed by the method of Redundancy Analysis (RDA).

Variables	Lambda-1^a^	Lambda-A^b^	*P* c	F d
CV of SBD	0.340	0.34	0.004	9.28
DBH	0.244	0.07	0.152	2.01
CV of soil TN	0.213	0.06	0.178	1.93
Soil pH	0.204	0.03	0.454	0.81
CV of pH	0.088	0.01	0.788	0.25

SBD: soil bulk density; DBH: diameter at breast height.

a Describe marginal effects, which shows the variance when the variable is used as the only factor.

b Describe conditional effects, which shows the additional variance each variable explains when it is included in the model.

c The level of significance corresponding to Lambda-A when performing Monte Carlo test (with 499 random permutations) at the 0.05 significance level.

d The Monte Carlo test statistics corresponding to Lambda-A at the 0.05 significance level.

## Discussion

### Variation in R_10_ and its determining variables

Soil respiration rate at 10°C has received little attention in contrast to the instantaneous rate of soil respiration in the study of soil carbon cycle. Moreover, the comparability of R_10_ under changing circumstances is more reasonable than that of normally measured soil respiration rate. Even at the same temperature of 10°C, soil basal respiration still exhibits a large variation within or across forest types. On the scale of the catchment, R_10_ varies in a range of 1.25 µmol CO_2_ m^−2^ s^−1^ to 2.30 µmol CO_2_ m^−2^ s^−1^ in the mixed forest with a mean value of 1.83 µmol CO_2_ m^−2^ s^−1^. R_10_ changes from 1.59 µmol CO_2_ m^−2^ s^−1^ to 2.46 µmol CO_2_ m^−2^ s^−1^ in the broadleaved forest with a mean rate of 2.01 µmol CO_2_ m^−2^ s^−1^. These values of R_10_ just fall well in the range of R_10_ of different forests in the same region [3], but they are slightly higher than those for pine and oak forests in Brasschaat [26]. Given that the similar empirical function has been applied in calculating the basal rate of soil respiration, the variation of R_10_ is greatly induced by environmental factors other than soil temperature.

Stand structure has been indicated to be a dominant factor accounting for the spatial variation in soil respiration in beech and mixed-dipterocarp forests. Basal area and DBH exert a significant positive effect on soil respiration [4], [27]. But, as to the specific results of this study, significant negative correlations are found between stand structure parameters and R_10_ for both forest types in the temperate region of North China. R_10_ declines differentially with the percentage of coniferous tree species in mixed forest community, and with the basal area across broadleaved forest stands. This intriguing scenario may be ascribed to the complexity of CO_2_ production in forest soils. Soil respiration consists of autotrophic respiration from roots and rhizosphere and heterotrophic respiration from microbial decomposition. Total rate and basal rate of soil respiration have been found to be slightly higher in the pure broadleaved forest stands than in the pure coniferous forest stands [3]. R_10_ is apparently depressed by the increasing admixed proportion of needle leaf tree species in the mixed forests. Perhaps, it is ascribed to the physiological differences between coniferous and broadleaved tree species. R_10_ is indicated to be modulated by plant photosynthesis (i.e. gross primary productivity) [9] via determining the activity of rhizosphere respiration [28]. Autotrophic respiration accounts for ∼50% of total soil respiration, which may even be higher in growing season for temperate forests [29]. In cold weather with temperature at ∼10°C, photosynthetic activity of the mixed forest stands with higher basal area can be heavily impeded, subsequently resulting in a lower R_10_.

Conversely, R_10_ significantly increases with the heterogeneous properties of shrub carbon stock and soil P content in the broadleaved forest stands and by the spatial variations in soil organic carbon content and TN content in the mixed forest stands. This may be due to the dominance of microbial respiratory fraction in total soil respiration at lower temperature. It is the microbial community composition and climatic factors that control forest soil respiration in cold seasons [30]. Additionally, soil microbial biomass and respiration have been eventually influenced by soil biophysical properties [31] and by environmental biochemical processes [32] through substrate availability, which indicates that soil respiration is essentially an enzymatic controlled process [9],[19].

### Variation in Q_10_ and its determinants

The temperature sensitivity of soil respiration, Q_10_, shows evident intra- and inter-forest-type variations on the catchment scale in temperate forests of northern China. The average Q_10_ values across forest stands for each forest type are larger than those of young plantations and a secondary *Populus davidiana* stand in the semiarid Loess Plateau [3]. However, the average Q_10_ is comparable to the reported values by Peng et al. [18] and by Zheng et al. [17] through synthesizing a great number of studies about temperature sensitivity of soil respiration on the spatial scales from region to country. In essence, variant Q_10_ values demonstrate the deficiency of the temperature dependent functions in describing the sensitivity of soil respiration to temperature.

The apparent Q_10_ derived from field experiment is actually a combined temperature sensitivity of different fractions of soil CO_2_ flux [10]. Particularly, the enzymatic reactivity of substrate decomposition to temperature is considered as the intrinsic Q_10_ [19]. Although the Q_10_ value of experimental study is suggested to be influenced by a wide range of ecological variables from molecular structure to climatic factors [18], [19], the direct determinant of the temperature sensitivity of soil respiration is still dependent on the substrate availability [19]. In this study, we find that Q_10_ could be markedly influenced by soil C/N ratio, soil pH, and the spatial heterogeneous properties of herbaceous carbon stock, SOC, soil TN, and soil bulk density. It is also worth mentioning that the contributors to the variations of Q_10_ differ considerably between these two forest types. Similar results have been reported that the Q_10_ values changed with the alteration of ecosystems and vegetation types [17], [18]. Indeed, the extrinsic factors pose the effect on temperature sensitivity mainly via the primary control of substrate availability.

It has been recognized that the assumption of constant Q_10_ of soil respiration is incorrect [9], [16], because the sensitivity of soil respiration to temperature is a complex reactivity; also it is more than being described by a simple parameter of temperature-dependent models. To date, a consensus has not been reached to clarify the mechanism underpinning the temperature sensitivity of soil respiration, but the study of easily monitored variables, such as soil physicochemical properties, SOC, soil TN, and forest type, etc., will help add extra predictive factors other than temperature in interpreting the variability of the apparent Q_10_.

### The effects of forest types on the correlations between biophysical variables and R_10_ and Q_10_

R_10_ and Q_10_ have been demonstrated by our results to be influenced by biophysical factors and their spatial variation in forest stands. Although similar intrinsic mechanisms account for the variations of R_10_ and Q_10_ with forest microenvironments, the specific determining factors of soil basal respiration still vary with forest type. This is because forest type consisting of different tree species displays great distinctions in biotic and abiotic variables, which ultimately manipulate the changing gradient and direction of R_10_ and Q_10_.

At our study site, the mixed forest type is mainly composed of the coniferous tree species *P*. *tabuliformis* and the broadleaved tree species *Q*. *wutaishanica*. *P*. *tabuliformis* in forest ecosystem perhaps takes responsibility for the distinct correlations of R_10_ and Q_10_ with biophysical variables between broadleaved and mixed forest types, because tree species determines not only the microbial community structure but also the decomposition dynamics of forest litter [33], [34]. Differences in the mycorrhizospheres and hyphospheres are the substantial way through which tree species affect soil microbial community including bacteria, archaea, fungi, and both free-living and symbiotic organisms [34]. Greater catabolic diversity and different bacterial and fungal communities have been found in the surface soil layer beneath mixed species plantations [35], and ectomycorrhizal fungi has been indicated to correlate with the presence of pine trees [36]. In addition, labile or soluble organic matter could also be affected by forest type with quantity and quality differences in litter and root exudates. This may induce the variations of soil microbial and enzyme activities between broadleaved and mixed forest types [37], [38]. Obviously, the anisotropic response of heterotrophic respiration derived from microbial activity to biophysical factors may account for the variant correlations of Q_10_ and R_10_ with measured variables between the two forest types.

In general, Q_10_ has a mathematical interrelationship with R_10_ and they also can be expressed by each other [10]. Furthermore, both Q_10_ and R_10_ display the confounding reactions of the complex process of soil respiration to the changes in exterior environmental factors. Therefore it can improve the overall understanding of the underlying mechanism driving soil respiration to concurrently analyze the variances of R_10_ and Q_10_ and their determinants. Although a single variable can explain larger variance of R_10_ or Q_10_, the comparatively lower attribution of the variation of Q_10_ and R_10_ to the five selected variables demonstrates the inherent interactions existing among biotic and abiotic variables. It is the internal interaction that determines the amplitudes of soil basal respiration rates with varying environmental conditions across temporal or spatial scales [10]. This viewpoint is also supported by the study of SØE and Buchmann [4]. Therefore, a more accurate estimation of soil CO_2_ efflux cannot be achieved for a specific forest ecosystem until the changes in Q_10_ and R_10_ are concurrently taken into account with alterations of microenvironment.
